# Loss of BRCA1-A Complex Function in RAP80 Null Tumor Cells

**DOI:** 10.1371/journal.pone.0040406

**Published:** 2012-07-06

**Authors:** Chunjing Bian, Rong Wu, Kathleen Cho, Xiaochun Yu

**Affiliations:** 1 Division of Molecular Medicine and Genetics, Department of Internal Medicine, University of Michigan Medical School, Ann Arbor, Michigan, United States of America; 2 Department of Pathology, University of Michigan Medical School, Ann Arbor, Michigan, United States of America; Harvard School of Public, United States of America

## Abstract

Receptor Associated Protein 80 (RAP80) is a subunit of the BRCA1-A complex and targets BRCA1 to DNA damage sites in response to DNA double strand breaks. Since mutations of BRCA1 are associated with familial ovarian cancers, we screened 26 ovarian cancer-derived cell lines for RAP80 mutations and found that TOV-21G cells harbor a RAP80 mutation (c.1107G >A). This mutation generates a stop codon at Trp369, which deletes the partial AIR region and the C-terminal zinc fingers of RAP80. Interestingly, both the mutant and wild type alleles of RAP80 lose their expression due to promoter hypermethylation, suggesting that TOV-21G is a RAP80-null cell line. In these cells, not only is the BRCA1-A complex disrupted, but the relocation of the remaining subunits in the BRCA1-A complex including BRCA1, CCDC98, NBA1, BRCC36 and BRE is significantly suppressed. Moreover, TOV-21G cells are hypersensitive to ionizing radiation, which is due to the compromised DNA damage repair capacity in these cells. Reconstitution of TOV-21G cells with wild type RAP80 rescues these cellular defects in response to DNA damage. Thus, our results demonstrate that RAP80 is a scaffold protein in the BRCA1-A complex. Identification of TOV-21G as a RAP80 null tumor cell line will be very useful for the study of the molecular mechanism in DNA damage response.

## Introduction

Ovarian cancer is the most frequent cause of cancer-related deaths among all gynecological cancers in the United States and is estimated to kill more than 140,000 women worldwide every year [1]. Like many other cancers, ovarian tumorigenesis is induced by genetic mutations. For example, nearly all high-grade serous ovarian carcinomas harbor *TP53* mutations [2]. In addition, approximately 10–15% of ovarian carcinomas occur in women with inherited mutations in *BRCA1* and *BRCA2*, who have an estimated 20–50% lifetime risk of ovarian carcinoma [3], [4].

BRCA1 is a tumor suppressor gene and is linked to various processes involved in the DNA damage response (DDR), including both repair of DNA double-strand breaks (DSBs) and cell cycle checkpoint control [5], [6]. The BRCA1 protein normally resides in nuclear multiprotein complexes and is the central component of at least three different complexes in humans, namely the A, B and C complexes [7], [8], [9], [10], [11], [12], [13]. These BRCA1 complexes function as signal transducers and repair effectors to correct abnormal DNA adducts at DSBs [14]. Thus, mutations of BRCA1 likely impair the repair of DNA lesions, thereby rendering the mutant cells prone to malignant transformation.

The BRCA1-A complex is the best-described and is involved in DNA repair via homologous recombination (HR) [15]. It contains ABRAXAS/CCDC98, RAP80, BRCC36, BRCC45/BRE, and NBA1/MERIT40/HSPC142 [8], [16], [17], [18], [19], [20], [21], [22]. In this complex, BRCA1 directly interacts with phospho-CCDC98. In response to DSBs, ATM phosphorylates histone H2AX, the variant of H2A, at the vicinity of DNA lesions. The phospho-H2AX then recruits MDC1 to DNA damage sites. Subsequently, ubiquitin E3 ligase RNF8 recognizes MDC1 and ubiquitinates histones at DNA damage sites with K63-linked poly-ubiquitin chains in the presence of E2 enzyme UBC13. The K63-linked poly-ubiquitin chains are then recognized by RAP80 via its ubiquitin interacting motif (UIMs). By interacting with CCDC98, RAP80 recruits BRCA1 to the DSBs for the next step in the DNA damage response [23].

Though a subset of breast and ovarian cancers are associated with germline BRCA1 or BRCA2 mutations, the majority are sporadic and do not have BRCA1 or BRCA2 mutations. Thus, it has been hypothesized that mutations of other functional partners in the BRCA pathway could account for a subset of breast and ovarian cancers lacking BRCA1 and BRCA2 mutations. Genetic studies have partially proven this hypothesis by identifying cancer-associated mutations in the BRCA1 and BRCA2 partners, such as BRIP1, PALB2 and Rad51 [10], [24], [25], [26], [27], [28], [29], [30], [31], [32]. However, it remains unclear whether mutation in the BRCA1-A complex is associated with tumorigenesis. Here, we conducted a mutational analysis of RAP80 in 26 ovarian cancer-derived cell lines and identified a truncating mutant of RAP80 in TOV-21G cells. Moreover, due to promoter hypermethylation, RAP80 is not expressed in TOV-21G cells, suggesting that TOV-21G is a RAP80-null cell line. In TOV-21G cells, not only the DNA damage-induced foci formation of the BRCA1-A complex is significantly suppressed, but also the BRCA1-A complex is disrupted, suggesting that RAP80 is the scaffold subunit in the BRCA1-A complex. Thus, our results not only reveal the *in vivo* function of RAP80, but also identify a RAP80 null ovarian cancer cell line, which will be very useful for studying the BRCA1-A complex-dependent DNA damage response.

## Results and Discussion

### Screening RAP80 mutations in human ovarian cancer cells

**Figure 1:**
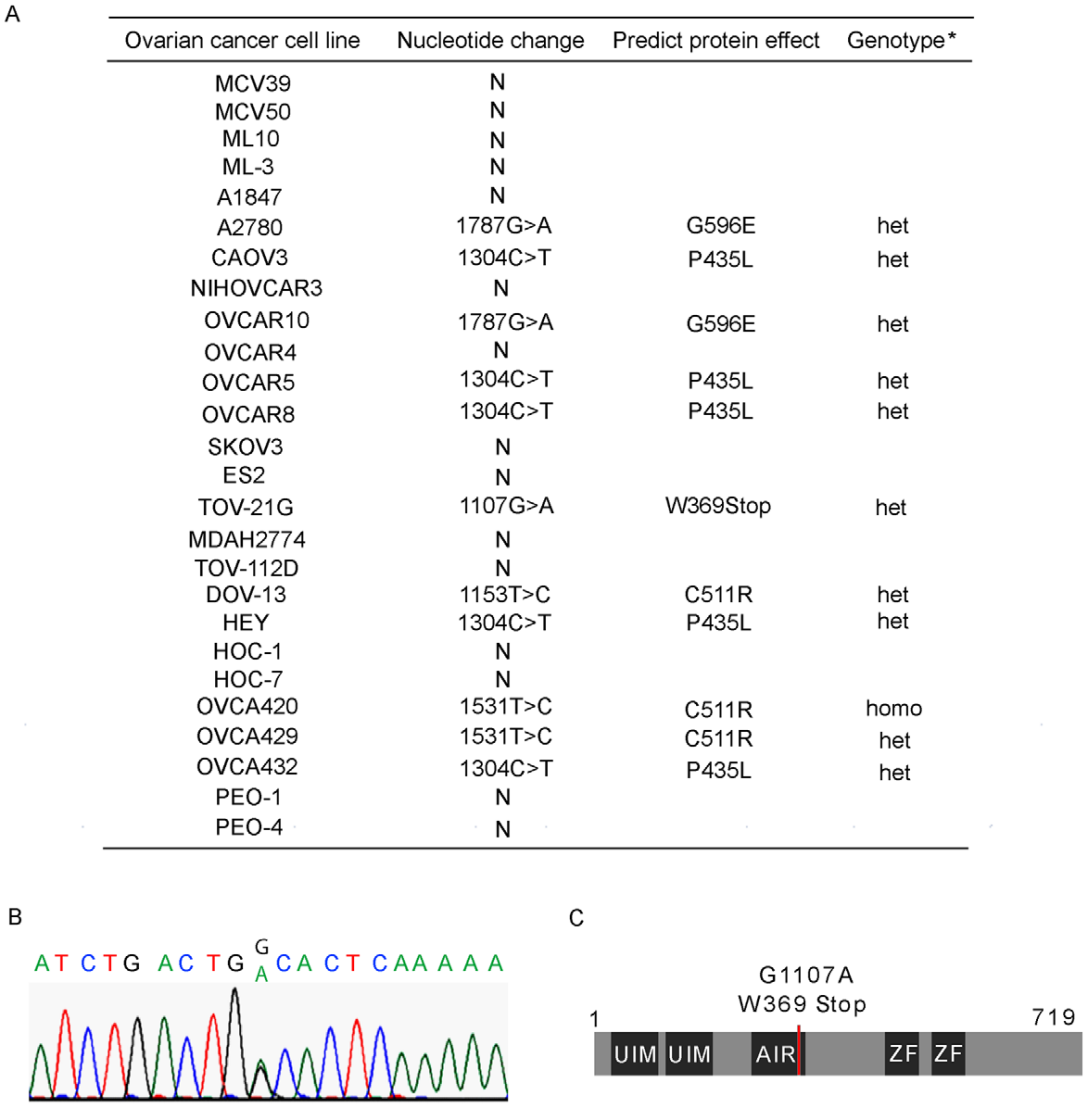
Screening RAP80 mutations in ovarian cancers. (A) RAP80 mutations in ovarian cancer cells. (B) The DNA sequences of mutant RAP80. (C) Sketch of RAP80 mutations.

To investigate whether RAP80 mutation is associated with ovarian tumorigenesis, we screened 26 human ovarian cancer cell lines for mutations in the coding sequences. The sequencing of RAP80 exons revealed a total of 4 different sequence variants (Figure 1A). According to the Uniprot database (http://www.uniprot.org/uniprot/Q96RL1), three of these alterations, including c.1304 C>T, c.1531 T>C and c.1787G>A, have been described as common polymorphisms and are unlikely to associate with susceptibility to ovarian cancer. The other variant, identified in TOV-21G cells, is c.1107G >A, which generates a stop codon at Trp369 and deletes the partial AIR region and the C-terminal zinc fingers of RAP80 (Figure 1B and C). The patient from whom TOV-21G was generated had been diagnosed with ovarian clear cell adenocarcinoma with wild type *TP53* [33].

### TOV-21G cell is a RAP80-null cell line

**Figure 2:**
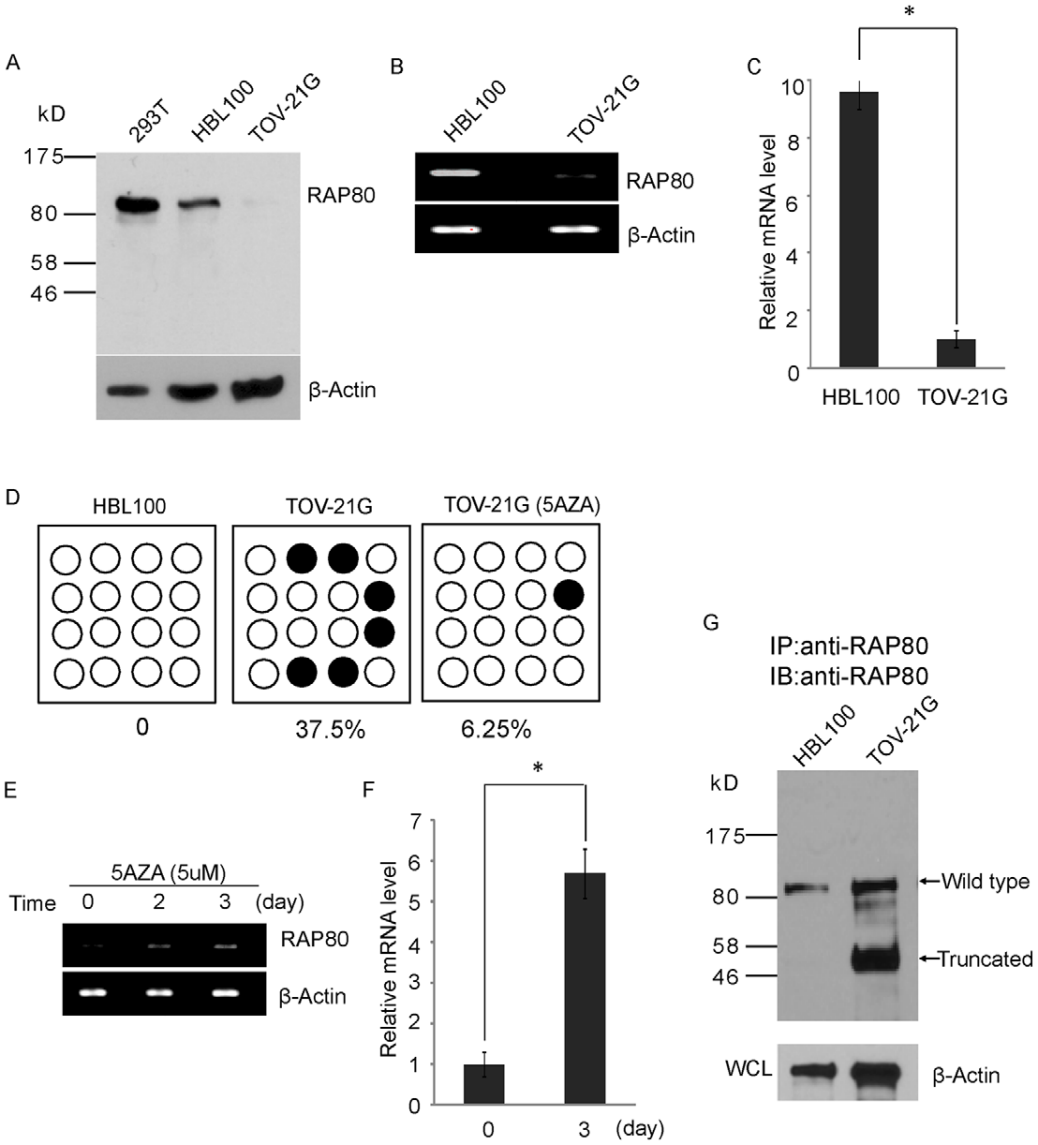
TOV-21G is a RAP80-null cell line. (A) The expression of RAP80 in 293T, HBL100 and TOV-21G cells was examined by Western blotting. β-actin was used as the protein loading control. (B) The transcription of *RAP80* gene was examined by RT-PCR. (C) Q-PCR shows the different mRNA level of RAP80 in HBL100 and TOV-21G cells. Results are shown as plus standard deviation (s.d.) from three independent experiments. *P values<0.01. (D) DNA methylation status of the CpG islands at the *RAP80* promoter region was examined by bisulfit sequencing. 5-AZA treatment significantly decreased the methylation level at the *RAP80* promoter in TOV-21G cells. (E, F) 5-AZA treatment increases the transcription of *RAP80* gene in TOV-21G cells. *P values<0.01. (G) 5-AZA treatment induces the protein expression of RAP80 in TOV-21G cells. The wild type and truncated mutant of RAP80 are indicated. β-actin was used as the protein loading control.

Due to the monoallelic mutation of *RAP80* gene in TOV-21G cells, we decided to examine the protein expression of both wild type and mutant RAP80 in this cell line. To our surprise, we could not detect the truncated RAP80 mutant by Western blotting. Moreover, although wild type RAP80 could be easily detected by Western blotting in 293T cells or HBL100 cells, a diploid epithelial cell line, the expression of RAP80 was undetectable in TOV-21G cells (Figure 2A). We also examined the expression of RAP80 in other 25 ovarian cancer cell lines by Western blotting. Again, only TOV-21G cells do not express RAP80 (Figure S1). Next, we examined the mRNA expression of RAP80 in TOV-21G. Based on RT-PCR, the level of RAP80 mRNA was extremely low in TOV21G cells relative to HBL100 cells (Figure 2B and C). Since loss of gene transcription is often induced by promoter hypermethylation, we examined the methylation status of CpG islands at the *RAP80* promoter region. Using software for predicting locations of CpG islands (http://cpgislands.usc.edu), a CpG islands cluster was predicted close to the transcription starting site (TSS) of the *RAP80* gene. Bisulfite sequencing analysis showed that 37.5% of CpG sites were methylated in TOV-21G cells, whereas few methylated CpG island was detected in HBL100 cell or other ovarian cancer cell lines (Figure 2D and Figure S2). Moreover, treatment with 5-AZA, an inhibitor of DMNT1, resulted in significantly reduced methylation of the CpG islands at the TSS of *RAP80* (Figure 2D). Correspondingly, mRNA transcription of *RAP80* gene was significantly increased following treatment with 5-AZA (Figure 2E and F). Using immunoprecipitation (IP) and Western blotting analysis, both wild type and truncated RAP80 mutant could be detected in TOV-21G cells following 5-AZA treatment (Figure 2E). Taken together, these results demonstrate that TOV-21G cell harbors a truncation mutation in *RAP80* gene. The expression of both wild type and mutant RAP80 is suppressed which is likely due to the promoter hypermethylation. In addition, although we did not examine other potential CpG islands, it is possible that other CpG islands methylation surrounding the TSS of *RAP80* gene or other abnormal epigenetic modifications may also contribute to silence *RAP80* gene in TOV-21G cells. Collectively, our results demonstrate that TOV-21G is a RAP80 null cell line.

### Ionizing radiation-induced foci formation (IRIF) of the BRCA1-A complex is suppressed in TOV21G cells

**Figure 3:**
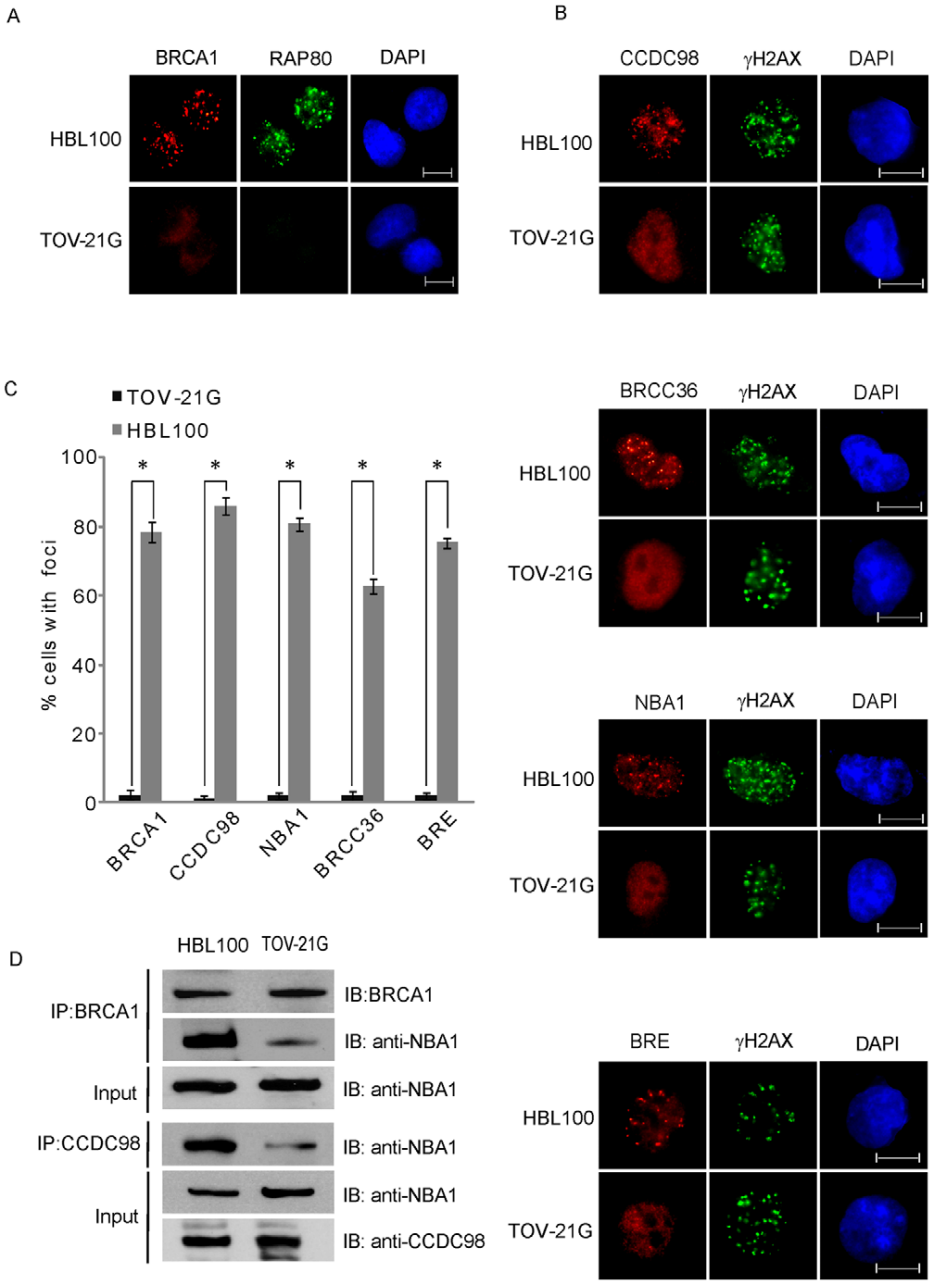
Loss of RAP80 suppresses the IRIF of the BRCA1-A complex. (A) The IRIF of BRCA1 is suppressed in TOV-21G cells. HBL100 and TOV-21G cells were treated with 10 Gy of IR. Cells were fixed and examined by indicated antibodies. Bar: 10 µm. (B) The IRIF of the BRCA1-A complex is impaired when loss of RAP80. The IRIF of endogenous CCDC98 and NBA1 was examined by indicated antibodies. The IRIF of BRCC36 and BRE was examined using cells stably expressing Flag-tagged BRCC36 and BRE. Bar: 10 µm. (C) Foci positive cells are summarized. Results are averaged (±s.d.) from three independent experiments. *P values<0.01. (D) The BRCA1-A complex is dissembled in TOV-21G cells. The interaction between endogenous BRCA1 and NBA1 and interaction between CCDC98 and NBA1 were examined by IP and Western blotting using indicated antibodies.

It has been reported that RAP80 is a subunit in the BRCA1-A complex and mediates the recruitment of BRCA1 to DNA damage sites [8], [17], [20]. Thus, using RAP80 null TOV-21G cells, we characterized the *in vivo* function of RAP80. We first examined the formation of ionizing radiation-induced foci (IRIF) of RAP80 and BRCA1 in TOV-21G cells. As shown in Figure 3A, the IRIF of both RAP80 and BRCA1 could be detected in HBL100 cells but not in TOV-21G cells, which is consistent with previous reports [8], [17], [20]. Besides BRCA1 and RAP80, the complex also has other subunits including CCDC98, BRCC36, NBA1 and BRE. We next examined the IRIF of these subunits in TOV-21G cells. As shown in Figure 3B, C and Figure S3, the IRIF of CCDC98, BRCC36, NBA1 and BRE were dramatically abrogated in the TOV-21G cells, suggesting that RAP80 is important for the relocation of the whole complex to DNA damage sites. In the BRCA1-A complex, it is known that RAP80 interacts with CCDC98 [8], [18], [22]. Next, we wondered whether the BRCA1-A complex is still intact in the absence of RAP80. Since NBA1 in the BRCA1-A complex mediates interactions between these subunits [16], [19], [21], we examined the interaction between CCDC98 and NBA1 and the interaction between BRCA1 and NBA1. The co-IP results demonstrate that these interactions were significantly impaired (Figure 3D), suggesting that RAP80 is important for the maintenance of the integrity of the BRCA1-A complex.

### RAP80 is involved in DNA damage repair

**Figure 4:**
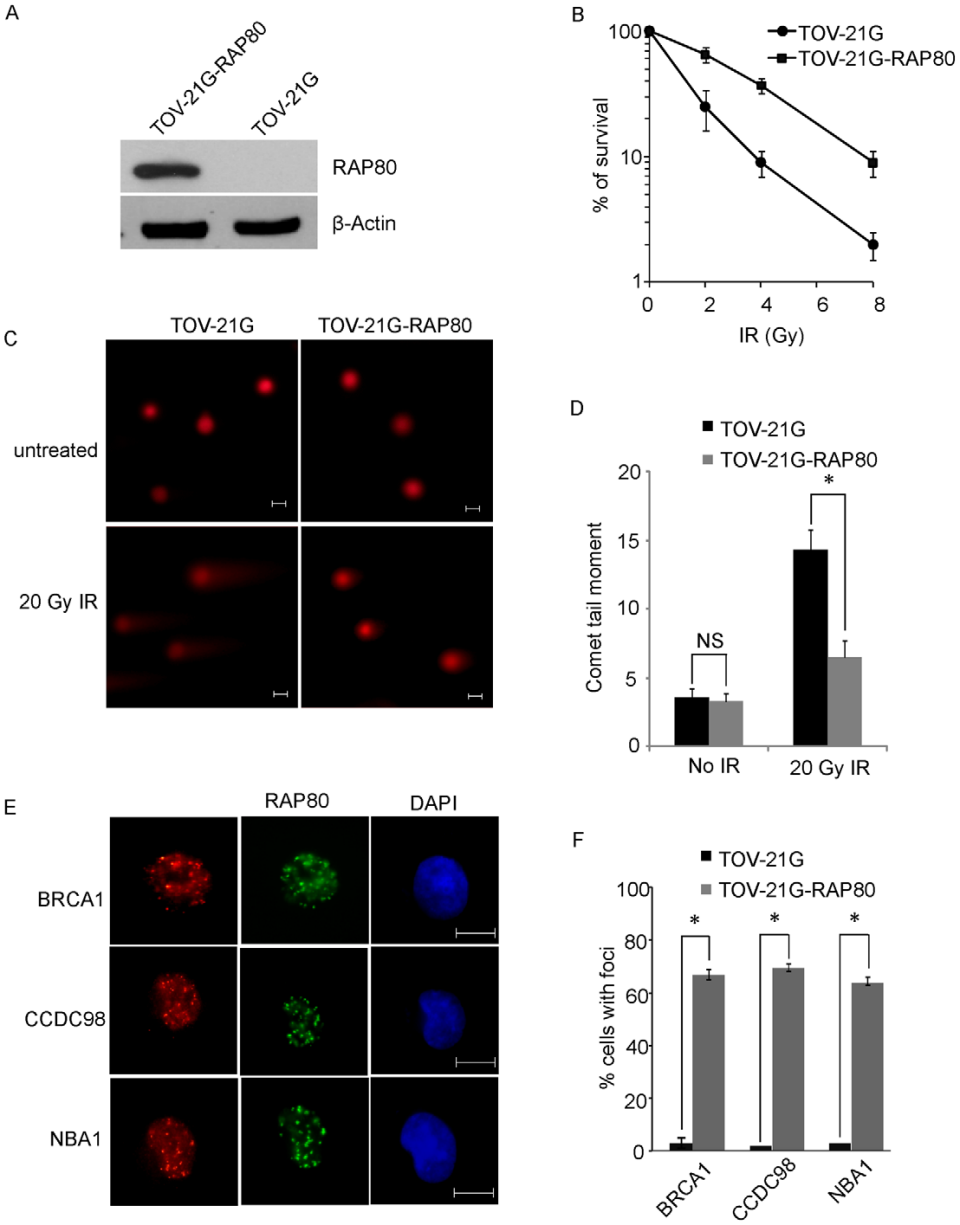
Loss of RAP80 abrogates DNA damage repair. (A) Expression of exogenous RAP80 in TOV-21G cells. (B) Cells loss of RAP80 are hypersensitive to IR. TOV-21G and TOV-21G-RAP80 cells were treated with indicated dose of IR. Survival cell colonies were calculated. (C) Comet assays show that loss of RAP80 impairs DNA damage repair. Representative images of neutral comet assays are shown. Bar: 10 µm. (D) The moment of comet tail were quantitatively measured. *P values<0.01; NS means no statistical significance. (E) Exogenous RAP80 restores the IRIF of the BRCA1-A complex in TOV-21G cells. Bar: 10 µm. (F) Foci positive cells are summarized. Data are from three independent experiments and error bars stand for standard deviation. *P values<0.01.

To study the functional significance of RAP80 in the DNA damage response *in vivo*, we performed clonogenic survival assays to examine whether RAP80 is important for cell viability in response to DNA damage. We first reconstituted TOV-21G cells with wild type RAP80 (Figure 4A). Compared to TOV-21G cells, TOV-21G-RAP80 cells with exogenous RAP80 expression were resistant to IR treatment (Figure 4B). It has been shown that BRCA1 plays an important role in DNA damage repair. Thus, the hypersensitivity to IR of TOV-21G cells is likely due to impaired DNA damage repair. Next, we used the “comet” assay to examine DNA damage repair in TOV-21G cells. With 20 Gy of IR treatment, unrepaired DNA fragments scored as the “comet tail” were observed in TOV-21G cells. However, in TOV-21G-RAP80 cells, DNA DSBs induced by IR treatment were significantly repaired. Thus, the “comet tail” was significantly reduced (Figure 4C and D). These results demonstrate that RAP80 is important for DNA damage repair. Moreover, in the presence of exogenous RAP80, the IRIF of BRCA1 and other subunits in the BRCA1-A complex was restored in TOV-21G cells, suggesting that RAP80 plays an important role in the DNA damage response (Figure 4E, F and Figure S4). We also examined the IRIF of the BRCA1-A complex when TOV-21G cells were treated with 5-AZA. With 5-AZA treatment, both wild type and mutant RAP80 were expressed (Figure 2G). Since the mutant also have the N-terminal UIM motifs that is critical for targeting RAP80 to DNA damage site [17], [20], the IRIF of RAP80 could be observed (Figure S5). However, the foci of BRCA1, CCDC98 and NBA1 were still abrogated in TOV-21G cells after IR treatment. It is possible that the mutant RAP80 acts as the dominant negative form to disrupt the BRCA1 A complex since the level of truncated RAP80 is much higher than that of wild type RAP80 (Figure 2G). But we could not rule out other possibilities since 5-AZA drastically alters a lot of gene expression. In this specific RAP80-null context, 5-AZA may also indirectly affect the BRCA1-A complex.

Taken together, our study identified a protein truncating mutation in one allele of RAP80 in TOV-21G cells. Expression of both RAP80 alleles in TOV-21G cells is silenced due to promoter hypermethylation, and hence, TOV-21G is a RAP80-null cell line. This is the first report of loss of RAP80 and functional BRCA1-A complex in ovarian cancer. Loss of RAP80 impairs the IRIF of the BRCA1-A complex and BRCA1-dependent DNA damage response, which could be a molecular mechanism contributing to ovarian tumorigenesis. Previous studies of familial breast cancers identified several missense mutations and a single amino acid deletion mutation of RAP80 [34], [35]. Although the functional consequences of these RAP80 mutations need to be further characterized, we and others have provided data supporting RAP80 as a bona fide breast and ovarian tumor suppressor.

We have shown that loss of RAP80 in TOV-21G cells abolishes the recruitment of the BRCA1-A complex to DNA lesions in response to DNA damage. Thus, BRCA1-dependent DNA damage repair is compromised in TOV-21G cells and the cells are hypersensitive to IR. Since TOV-21G cells lose the functional BRCA1-A complex, this cell line could be very useful for DNA damage research as well as screening new drugs for treating familial breast and ovarian cancers.

## Materials and Methods

### Ovarian cancer cell lines

The 26 ovarian cancer cell lines are listed in Figure 1A. ML3 and ML10 are cystadenoma-derived cell Lines, which were generated in Dr. Louis Dubeau lab [36]. Cell lines MCV39 and MCV50 are derived from ML10 [37]. A2780 and A1847 are epithelial ovarian cancer cell lines that were derived from patients prior to treatment [38]. The ES-2 cell line has originally been derived from a poorly differentiated ovarian clear cell carcinoma with fibroblast morphology. CAOV3, NIHOVCAR-3, SKOV3, MDAH2774 have been cultured from malignant ascites from patients with adenocarcinoma. HOC-1, HOC-7 and HEY are ovarian serous carcinoma cell lines [39]. OVCA420, OVCA429, and OVCA432 cell lines were established from freshly isolated ascites or tumor explants from patients with late-stage ovarian adenocarcinomas with distinct characteristics [40]. Two ovarian cancer cell lines (PEO1 and PEO4) were derived from a *BRCA2* mutation [5193C>G (Y1655X)] carrier with ovarian carcinoma, which acquired cisplatin resistance and a secondary *BRCA2* mutation [5193C>T (Y1655Y)] that canceled the inherited mutation [41].

### Mutation Analysis

The complete coding sequence of RAP80 (NM 016290) was analyzed for genetic alterations in all cell lines. All unique sequence alterations were confirmed by sequencing of an independently amplified template. Primer sequences are listed in the Table S1.

### Antibodies, plasmids and cell cultures

Rabbit anti-RAP80, CCDC98 antibodies were described previously. N-terminal of RAP80 (residues 1–354) was expressed as GST-RAP80 to immunize rabbit and generate polyclonal antibody against RAP80 [18]. Rabbit anti-NBA1 antibody was generated by immunizing rabbit with GST-NBA1. Mouse anti-BRCA1 monoclonal antibody (SD118) was purchased from Oncogene. Rabbit anti-BRCA1 polyclonal antibody was purchased from Upstate. Anti-Flag (M2) antibody and mouse anti-β-actin monoclonal antibody (AC-15) were from Sigma. Mouse anti-γH2AX monoclonal antibody (JBW301) was from Upstate.

TOV-21G, HBL100 and 293 T cells were cultured in Dulbecco's Modified Eagle Medium with 10% (v/v) FBS. To establish stable cell lines expressing RAP80, TOV-21G cells were transfected plasmids encoding SFB-RAP80 using Lipofectamine2000 (Invitrogen). Transfected cells were subsequently selected in the presence of G418 (600 μg/ml) for 5 weeks. The expression of RAP80 in TOV-21G cells was determined by Western blotting using anti-RAP80 antibody.

For IR treatment, cells were irradiated using a JL Shepherd ^137^Cs radiation source with indicated doses and then recovered under the same culture condition for further analysis.

For 5-AZA treatment, cells were treated with 5 µM 5-AZA for 3 days, and the fresh medium with 5-AZA was used to culture cells every day.

### Cell lysis, immunoprecipitation and Western blotting

Cells were lysed with NETN100 buffer (0.5% Nonidet P-40, 50 mM Tris-HCl pH 8.0, 2 mM EDTA, and 100 mM NaCl) unless otherwise specified. Immunoprecipitation and Western blotting were performed following standard protocol as described anywhere else. For the analysis of BRCA1 expression, the whole-cell lysates from HBL100 and TOV-21G cells were subjected to IP using anti-BRCA1 polyclonal antibody and Western blotting using anti-BRCA1 monoclonal antibody.

### RT-PCR

The mRNA expression level of RAP80 was measured by RT-PCR as described anywhere else. Quantitative-PCR was performed using Power SYBR green PCR master mix in 7300 real time PCR systems (Applied Biosystems). The mean value was calculated by three independent experiments. The primers used in this experiment are list in Table S2.

### Bisulfite sequencing

The CpG island methylation level was measured by bisulfate sequencing. For optimized bisulfite conversion, we employed 500 ng of genomic DNA and followed the manufacturer's protocol of the Zymo EZ DNA Methylation Kit (Kit #D5001). The bisulfite-treated DNA was subjected to nesting PCR. The primers are listed as follows. The outside primers are 5′-ATTATTGGTGTTGGGTTGGGTTTTTTTAG-3′ and 5;-TTCAACTACAAAACACTACCTCCCTTAAC-3′. The inside primers are 5′-GTTAGGTTGGGTTTTGTATTTTTGGTTG-3′ and 5′-CTACTAAAATTCAAATCCAACTCCACTAC-3′. The PCR products were separated by 1% agarose gel, purified by gel extraction kit and then cloned to T vector for sequencing.

### Immunofluorescence staining

To visualize IR-induced foci, cells were grown on coverslips and treated with 10 Gy of IR followed by 6 hours' recovery, then cells were fixed with 3% paraformaldehyde at room temperature for 30 minutes, and permeabilized with PBS containing 0.5% Triton X-100 at room temperature for 15 minutes. The cells were then blocked with PBS containing 3% goat serum at room temperature for 30 minutes and primary antibodies at room temperature for one hour. After washing three time with PBS, cells were incubated with secondary antibody, FITC- conjugated goat anti-mouse IgG, FITC-conjugated goat anti-rabbit IgG, rhodamine-conjugated goat anti-rabbit IgG, or rhodamine-conjugated goat anti-mouse IgG (Jackson Immuno-Reserach Laboratories, Inc.) at room temperature for 30 minutes. Cell nuclei were then counterstained with 4, 6-diamidino-2-phenylindole (DAPI). After a final wash with PBS, coverslips were mounted with glycerin containing p-phenylenediamine. All images were obtained with an OLYMPUS IX71 fluorescence microscope. For each sample, we examined 1000 cells. Since spontaneous foci exist in untreated cells, cells with more than 5 foci in each nucleus were considered as foci positive cells following IR treatment.

### Cell survival assays

Five hundred TOV-21G or RAP80-reconstituted TOV-21G cells were plated in the wells of a 6-well plate immediately following 2, 4 or 8 Gy of IR. After incubation for 10 days, the surviving cell fractions were calculated by comparing the numbers of colonies formed in the irradiated cultures with those in unirradiated controls.

### Neutral comet assay

Single-cell gel electrophoretic comet assays were performed under neutral conditions [42]. Briefly, TOV-21G and TOV-21G-RAP80 cells were irradiated with 20 Gy and incubated in culture medium at 37°C for 4 hours. For cellular lysis, the slides were immersed in neutral lysis solution (2% sarkosyl, 0.5 M EDTA, 0.5 mg/ml proteinase K, pH 8.0) overnight at 37°C. On the second day, after electrophoresis at 15 V for 25 minutes (0.6 V/cm), the slides were stained for 20 minutes with 10 μg/ml propidium iodide and viewed in a fluorescence microscope. The comet tail moment was analyzed by CometScore software.

## Supporting Information

Figure S1 **The expression of RAP80 in 26 ovarian cancer cell lines was examined by Western blotting.** β-actin was used as the protein loading control.
(TIF)

Figure S2 **DNA methylation status of the CpG islands at the** ***RAP80*** **promoter region was examined by bisulfite sequencing in TOV-112D, SKOV3, DOV-13, OVCAR10 and TOV-21G cells.**
(TIF)

Figure S3 **The IRIF of the BRCA1-A complex is impaired when loss of RAP80.** The IRIF of endogenous CCDC98 and NBA1 was examined by indicated antibodies. The IRIF of BRCC36 and BRE was examined using cells stably expressing Flag-tagged BRCC36 and BRE. Bar: 10 µm.
(TIF)

Figure S4 **Exogenous RAP80 restores the IRIF of the BRCA1-A complex in TOV-21G cells.** Bar: 10 µm.
(TIF)

Figure S5 **5-AZA treatment only restores the IRIF of RAP80 but not the IRIF of other subunits in the BRCA1-A complex.** Bar: 10 µm.
(TIF)

Table S1 **Primer sequences were used for amplication of RAP80 exons.**
(DOC)

Table S2 **Nucleotide sequences of the RT-PCR primers.**
(DOC)
