# Supplementary figures and images for: Loss of BRCA1-A Complex Function in RAP80 Null Tumor Cells
Source: PLoS One. 2012 Jul 6;7(7):e40406. doi: 10.1371/journal.pone.0040406 (PMC3391255; doi:10.1371/journal.pone.0040406)

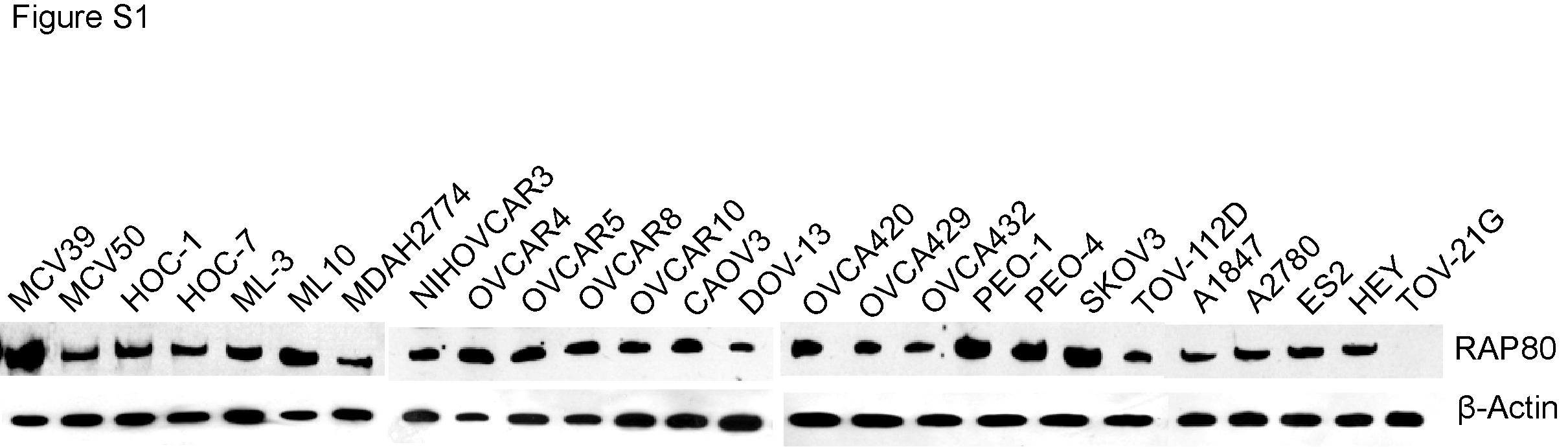

Supplement: Figure S1 — The expression of RAP80 in 26 ovarian cancer cell lines was examined by Western blotting. β-actin was used as the protein loading control. (TIF) [file pone.0040406.s001.tif]

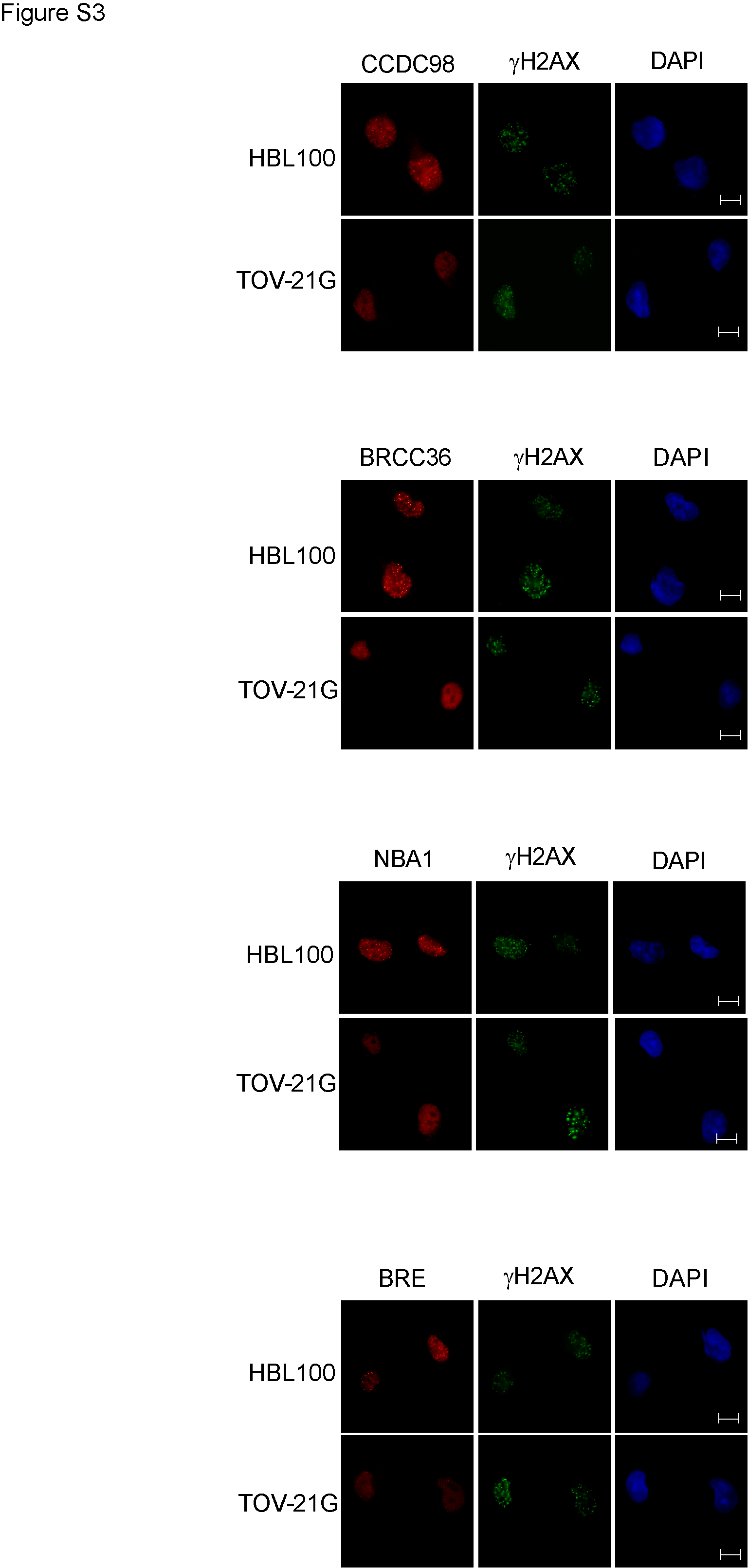

Supplement: Figure S3 — The IRIF of the BRCA1-A complex is impaired when loss of RAP80. The IRIF of endogenous CCDC98 and NBA1 was examined by indicated antibodies. The IRIF of BRCC36 and BRE was examined using cells stably expressing Flag-tagged BRCC36 and BRE. Bar: 10 µm. (TIF) [file pone.0040406.s003.tif]

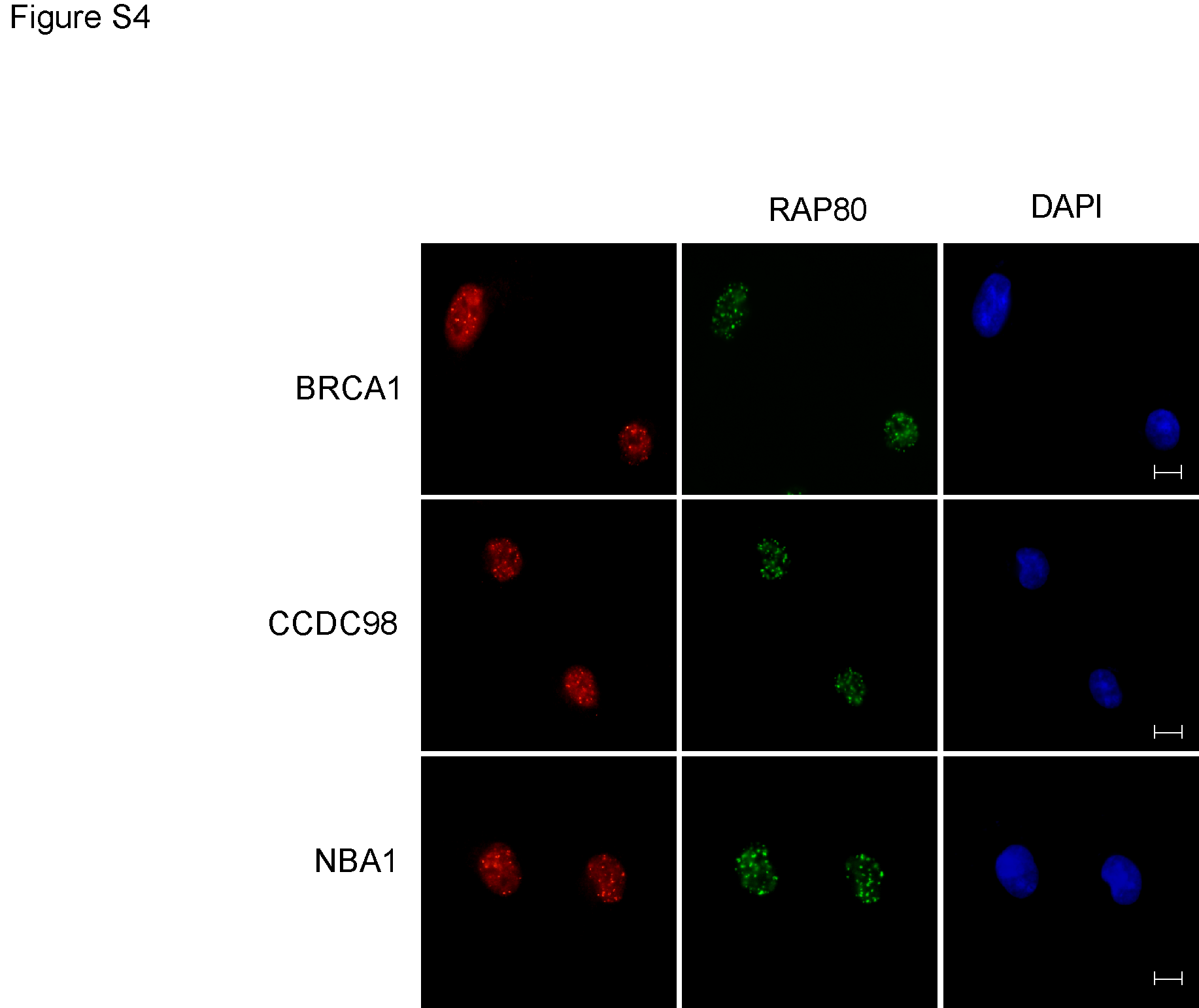

Supplement: Figure S4 — Exogenous RAP80 restores the IRIF of the BRCA1-A complex in TOV-21G cells. Bar: 10 µm. (TIF) [file pone.0040406.s004.tif]

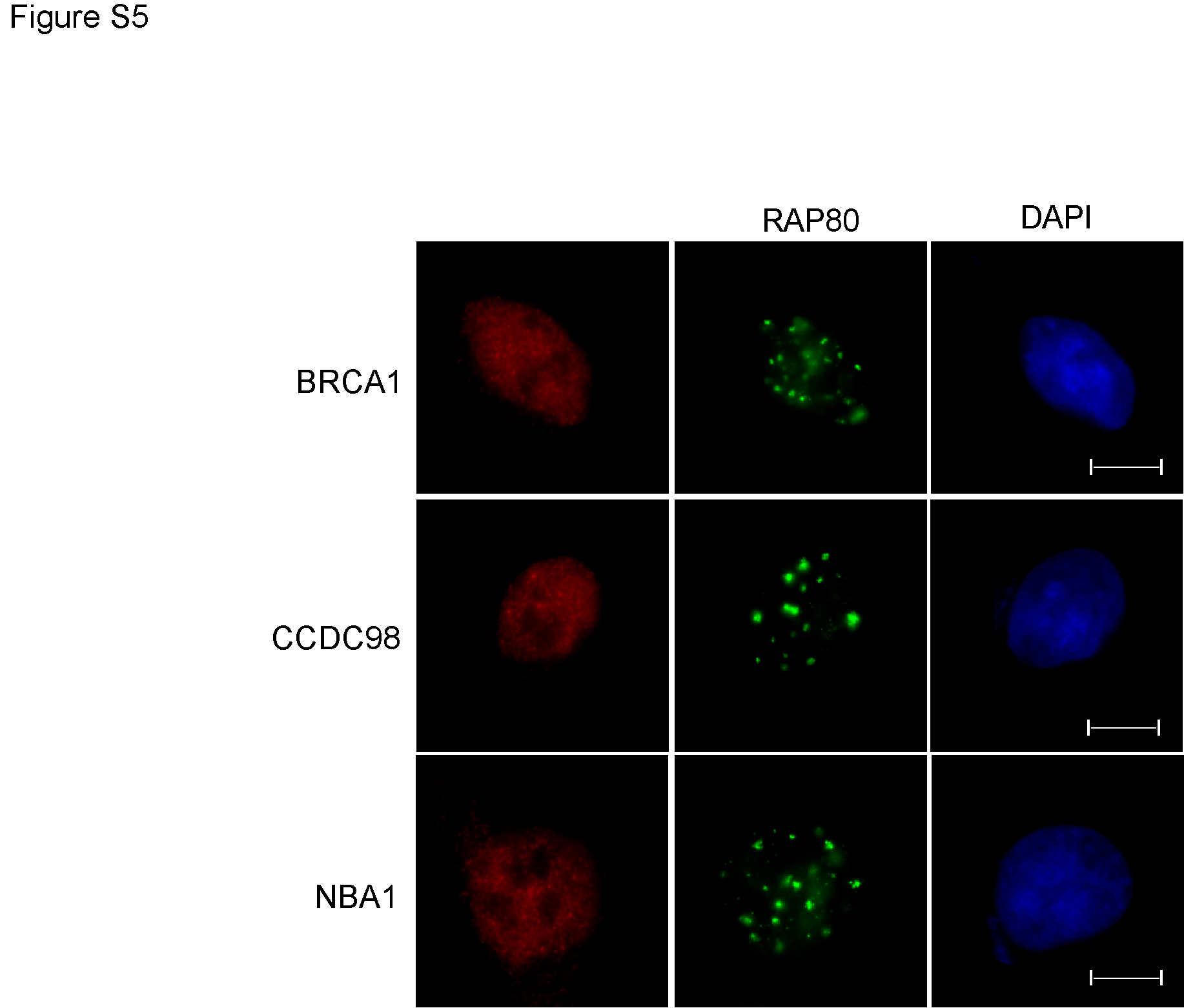

Supplement: Figure S5 — 5-AZA treatment only restores the IRIF of RAP80 but not the IRIF of other subunits in the BRCA1-A complex. Bar: 10 µm. (TIF) [file pone.0040406.s005.tif]
