# Supplementary material for: Loss of BRCA1-A Complex Function in RAP80 Null Tumor Cells
Source: PLoS One. 2012 Jul 6;7(7):e40406. doi: 10.1371/journal.pone.0040406 (PMC3391255; doi:10.1371/journal.pone.0040406)
Supplement: Table S1 — Primer sequences were used for amplication of RAP80 exons. (DOC) [file pone.0040406.s006.doc]

| **Table S1 Primer sequences were used for amplication of RAP80 exons** | | |
| --- | --- | --- |
|  |  |  |
| **Exon** | **Sequence (5'-3')** | **Tm (°C)** |
| 1 | Forward GGCAACAAAGCGAGACCATCTCAA | 57.3 |
|  | Reverse GGCATTAAGGTGAAGTCAGTCACG | 57.3 |
| 2 | Forward AATCACCAATTCCTCAGAGGCCTC | 57.3 |
|  | Reverse GAATCTATCAGAGACCTAAGAATGCAAC | 56.9 |
| 3 | Forward GTGGTCTGGTAGTGTCTTCAGTTG | 57.3 |
|  | Reverse TACAGCTAGTTCCTAATGCAACTACAAC | 56.9 |
| 4 | Forward CCTCCCATTTTGTCCTTCCTGTGA | 57.3 |
|  | Reverse AGCATGAGATCCCTGTGGTACAGT | 57.3 |
| 5a | Forward CCCAAAGAAGAGCCTTTCTTACCC | 57.3 |
|  | Reverse AGGGCAGAATGGAATACCCCAGAA | 57.3 |
| 5b | Forward TAGCAGATGCCAAAGGTCTCCAG | 56.9 |
|  | Reverse TAATAATCAAGGAGGGGAAGACCACAA | 56.6 |
| 6 | Forward AGGAAGAGAAGCCAGTTCACCTGT | 57.3 |
|  | Reverse TTATGCTGGCAACCATCAGAGGAG | 57.3 |
| 7 | Forward GGCTTTGTGAGTGACTCACCATTG | 57.3 |
|  | Reverse CCTCCAGAGAGTAGGTTTGAAGAG | 57.3 |
| 8 | Forward ATATTGCCACCACAGTATTGTCATATCC | 56.9 |
|  | Reverse TCAACGTTGCATCAGAGAGAGGAC | 57.3 |
| 9 | Forward GGAGTCTCTTGCAGTTCGTTGTAG | 57.3 |
|  | Reverse AGTAAGAGTTTGGCTGTCCTGCTG | 57.3 |
| 10 | Forward CAGAGTCTACTGGCAGCTAACTATG | 57.6 |
|  | Reverse ATACAAGAGCTCCGTGAAGCTAGC | 57.3 |
| 11 | Forward GCCCAGAGGCAAAAGCACATCTTA | 57.3 |
|  | Reverse CCTCTTACAACTGACAACCAGGAG | 57.3 |
| 12 | Forward TGTGATCTGGGCTGTGGAAGTTGT | 57.3 |
|  | Reverse TCTTCACTGAAAGGCCTTAGGCAG | 57.3 |
| 13 | Forward CCTAGAGCACGGAAGCAAAAGATC | 57.3 |
|  | Reverse AGGACAAGGCCTGCACTTTTCAGA | 57.3 |
| 14 | Forward GCATGTCCTTGATGCTTAGTAGGTG | 57.6 |
|  | Reverse CACCCCTCCTACTAATGGTTTTGTC | 57.6 |
