# Supplementary material for: Loss of BRCA1-A Complex Function in RAP80 Null Tumor Cells
Source: PLoS One. 2012 Jul 6;7(7):e40406. doi: 10.1371/journal.pone.0040406 (PMC3391255; doi:10.1371/journal.pone.0040406)
Supplement: Table S2 — Nucleotide sequences of the RT-PCR primers. (DOC) [file pone.0040406.s007.doc]

| **Table S2 Nucleotide sequences of the RT-PCR primers** | |  |
| --- | --- | --- |
|  |  |  |
| **Gene** | **Sequence (5'-3')** | **Fragment size (bp)** |
| RAP80 | Forward: GTGATATCCGATAGTGATGGAGAGG | 277 |
|  | Reverse: GAGGTCGAGATCTGGTAGCGGAAGC |  |
| β-actin | Forward: AAAGACCTGTACGCCAACAC | 220 |
|  | Reverse: GTCATACTCCTGCTTGCTGAT |  |
